# A Systematic Analysis of Candidate Genes Associated with Nicotine Addiction

**DOI:** 10.1155/2015/313709

**Published:** 2015-05-04

**Authors:** Meng Liu, Xia Li, Rui Fan, Xinhua Liu, Ju Wang

**Affiliations:** School of Biomedical Engineering, Tianjin Medical University, 22 Qixiangtai Road, Tianjin 300070, China

## Abstract

Nicotine, as the major psychoactive component of tobacco, has broad physiological effects within the central nervous system, but our understanding of the molecular mechanism underlying its neuronal effects remains incomplete. In this study, we performed a systematic analysis on a set of nicotine addiction-related genes to explore their characteristics at network levels. We found that NAGenes tended to have a more moderate degree and weaker clustering coefficient and to be less central in the network compared to alcohol addiction-related genes or cancer genes. Further, clustering of these genes resulted in six clusters with themes in synaptic transmission, signal transduction, metabolic process, and apoptosis, which provided an intuitional view on the major molecular functions of the genes. Moreover, functional enrichment analysis revealed that neurodevelopment, neurotransmission activity, and metabolism related biological processes were involved in nicotine addiction. In summary, by analyzing the overall characteristics of the nicotine addiction related genes, this study provided valuable information for understanding the molecular mechanisms underlying nicotine addiction.

## 1. Introduction

Cigarette smoking is the most common form of tobacco use and is one of the main preventable causes of premature death and disability worldwide [1, 2]. Although there are some effective control policies and interventions on tobacco abuse, the negative impact of tobacco dependence on society is still staggering. The World Health Organization estimates that there are currently about 1.3 billion smokers worldwide, resulting in approximately 5 million annual tobacco attributable deaths [3, 4]. If the current trend continues, by 2020, smoking will become the largest single health problem worldwide, causing 10 million deaths annually, mostly in low- and middle-income countries [5]. Despite these grim statistics, cigarette smoking continues to impose substantial health and financial costs on society. According to the Centers for Disease Control and Prevention (CDC), in USA alone, the economic burden caused by smoking to society, including both the direct health care expenditures and the loss of productivity, can be as high as $193 billion a year [6]. In china, the prevalence of smoking remains high with 350 million smokers, and it is estimated that, by 2025, the annual number of deaths attributed to tobacco use will increase from 1.2 million to 2 million [7]. Although many cigarette smokers report a desire to quit smoking [8], few are successful [9, 10]. Thus, developing effective therapeutic approaches that can help smokers achieve and sustain abstinence from smoking, as well as methods that can prevent people from starting smoking, remains a huge challenge in public health.

Nicotine, as the primary psychoactive component of tobacco smoke, produces diverse neurophysiological, motivational, and behavioural effects through interactions with nicotinic acetylcholine receptors (nAChRs) in the central nervous system (CNS). Twins, family and adoption studies have suggested that nicotine addiction is closely related to genetic and environmental factors, and genetic factors play an important role in the risk to the development of addiction [11, 12]. Numerous studies aiming to identify the genetic variants or candidate genes have found a large number of promising genes and chromosomal regions involved in the etiology of nicotine addiction [13]. In addition, various pathways and neurotransmitter systems have been found to be related to the psychoactive and addictive properties of nicotine, such as the mesocorticolimbic dopamine system [14–16], the serotonin system, the glutamate system, and the GABA system [17–19]. Further, emerging evidence suggests that nicotine can also regulate the expression of genes/proteins involved in various functions such as ERK1/2, CREB, and c-FOS [20–22], as well as the expression state of multiple biochemical pathways, for example, mitogen-activated protein kinase (MAPK), phosphatidylinositol phosphatase signaling, growth factor signaling, and ubiquitin-proteasome pathways [23–25].

During the past decade, the application of high-throughput technologies to nicotine addiction study has greatly enhanced our ability to identify the nicotine addiction-related molecular factors [26–28]. In spite of these progresses, our understanding of the molecular mechanism underlying nicotine addiction is still incomplete. Under such situation, how to integrate the available knowledge and data in heterogeneous datasets to obtain the relevant biological information has become an important task. Among the available approaches to explore the molecular mechanisms underlying various complex diseases, investigating the interactions between proteins encoded by the candidate genes in the human protein-protein interaction (PPI) network has been emerging as a powerful way [29–31]. Furthermore, genes/proteins with similar functions usually interact with each other more closely than those functionally unrelated genes [32], and cluster analysis on the molecular candidates within a PPI network can provide an intuitive view to understand its major biological functions. Taking together, a comprehensive analysis of the candidate genes within a systematic framework may be a powerful approach to analyze the molecular mechanisms underlying complex diseases like nicotine addiction.

In this study, the global network topological properties of nicotine addiction-related genes (NAGenes) were explored in the context of human PPI network and were compared with other gene sets. Then, cluster analysis was utilized to detect the major functional modules related to nicotine addiction in the PPI network. Additionally, the significantly enriched functional clusters were identified for the NAGenes. This study provides useful insights for understanding the molecular mechanisms of nicotine addiction at the systems biological level.

## 2. Materials and Methods

### 2.1. Data Sources

Multiple gene sets related to nicotine abuse have been reported [27, 33, 34]. In an earlier study, we obtained 220 NAGenes prioritized via a multisource-based gene approach [35], which represented a relatively comprehensive gene set for nicotine addiction. Briefly, genes identified to be related to nicotine addiction or involved in the physiological response to nicotine exposure or smoking behaviors were collected by integrating four categories of evidence, that is, association studies, linkage analysis, gene expression analysis, and literature search of single gene/protein-based studies. A category-specific score was assigned to each gene and a combined score was computed for all the collected genes based on an optimized weight matrix. Then, the genes were ranked according to the combined scores with a larger score value indicating a potentially higher correlation between the gene and nicotine addiction. Based on the distribution of the combined score of all the genes collected, 220 genes on the top of the list were selected as the prioritized NAGenes.

For the purpose of comparison, we collected two other gene sets, that is, an alcohol addiction-related gene set (alcohol genes) and a cancer-related gene set (cancer genes). Alcohol addiction can evoke the dysfunction of neuronal system and has been suggested to share some biological mechanisms with nicotine addiction. In this study, we selected the gene set with 316 alcohol genes collected by Li et al. [33]. Cancer has been well studied and is expected to have substantially different pathological characteristics from nicotine addiction. We downloaded the cancer genes (522 genes) from the Cancer Gene Census database (http://cancer.sanger.ac.uk/cancergenome/projects/cosmic/).

To investigate the network topological characteristics of a gene set, we first need to construct a relatively comprehensive and reliable PPI network. Here, we downloaded the human PPI data from the Protein Interaction Network Analysis (PINA) platform (May 21, 2014) [36], which collected and annotated data from six major protein interaction databases, that is, IntAct, BioGRID, MINT, DIP, HPRD, and MIPS/MPact. Also, we downloaded several related annotation files from NCBI (ftp://ftp.ncbi.nlm.nih.gov/gene/) (May 24, 2014), including the Entrez gene information database of human (Homo_sapiens.gene_info.gz), the data set specifying relationship between pairs of NCBI and UniProtKB protein accessions (gene_refseq_uniprotkb_collab.dz), and file containing mappings of Entrez Gene records to Entrez RefSeq Nucleotide sequence records (gene2refseq.gz). For the proteins included in the human PPI database, only those that could be mapped to NCBI Entrez Gene were included in our subsequent analysis. After excluding the redundant and self-interacting pairs, we constructed a human PPI network containing 15,093 nodes and 161,419 edges.

### 2.2. Global Network Topological Properties

In network analysis, different metrics can be used to describe the network characteristics. We applied four measures to assess the network topological characteristic of NAGenes, that is, degree and degree distribution, clustering coefficient, closeness, and eccentricity. For a network, degree of a node (gene/protein in our case) is the number of direct connections that it has to other nodes in the network, and highly linked nodes are usually thought to make important contribution to the global structure or the behavior organization of a biological network [37, 38]. Degree distribution is the probability distribution of the degrees of all nodes over the whole network. Clustering coefficient quantifies the probability that two nodes linking to the same node connect with each other and describes the overall organization of the relationships within a network [39, 40]. The closeness of a node is the reciprocal of its average distance to each node in the network, while the eccentricity of a node is the distance to its farthest reachable node [41].

### 2.3. Cluster Analysis within the Global Network

To intuitively observe the biological functions involved in the large nicotine addiction-related network, we applied the Molecular Complex Detection (MCODE) (Version 1.4) (http://baderlab.org/Software/MCODE) implemented in Cytoscape platform (http://www.cytoscape.net/) to identify the molecule modules or clusters. MCODE is a local clustering algorithm that can effectively detect densely connected regions of a molecular interaction network. In our analysis, the global network that we constructed was uploaded into the Cytoscape [42] and then MCODE was run to detect gene clusters in the network using the haircut option which identified nodes having limited connectivity at the cluster periphery. For the other parameters, the default settings were adopted.

### 2.4. Functional Annotation Cluster

To assess the candidate genes in the context of function similarity, we performed enrichment analysis on their Gene Ontology (GO) annotations using the Database for Annotation and Integrated Discovery (DAVID) (Version 6.7) [43]. The genes with their gene ID or GenBank Accession Numbers were submitted to DAVID under the functional annotation option specifying Homo sapiens as the species. In the DAVID functional annotation clustering, the significantly overrepresented GO terms, that is, biological process (BP), molecular function (MF), and cellular component (CC), were retrieved by using the options GOTERM_BP_ALL, GOTERM_MF_ALL and GOTERM_CC_ALL. The default parameters and corresponding false discovery rate (FDR) by the Benjamini and Hochberg approach [44] were used to determine the enrichment score.

## 3. Results and Discussions

### 3.1. Global Network Topological Properties of NAGenes

PPI network analysis provides an effective approach to investigate the biological themes related to a list of genes at the molecular level. In particular, the topological properties of nodes (genes) and edges (connections between genes) can help to understand the underlying biological themes associated with the network [45]. To depict the network topological properties of NAGenes, we first constructed a human PPI network by integrating information from multiple databases, to which NAGenes were then mapped. Subsequently, the characteristics of the NAGenes in the network were assessed by four network topological measurements, that is, degree, clustering coefficient, closeness, and eccentricity. As a comparison, we also calculated the topological measures of the networks corresponding to alcohol genes and cancer genes.

**Figure 1:**
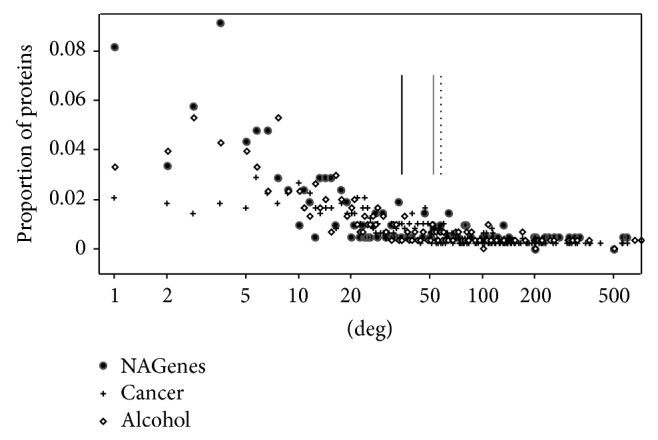
Degree distribution and the average degree of NAGenes, alcohol genes, and cancer genes. *y*-axis represents the proportion of proteins having a specific degree. Vertical line represents the average value of the degrees. Black line denotes NAGenes, gray line denotes alcohol genes, and dotted line denotes cancer genes.

**Figure 2:**
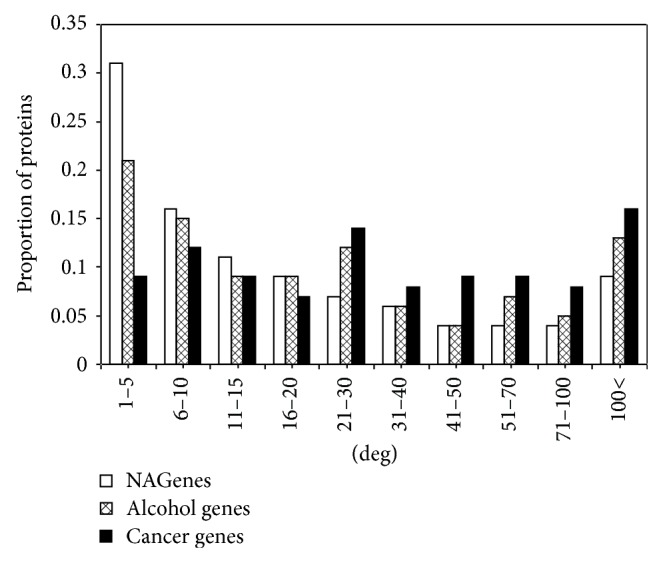
Degree distribution of NAGenes, alcohol genes, and cancer genes. *y*-axis represents the proportion of proteins having a specific degree.

Of the 220 NAGenes, 208 could be mapped onto the human PPI network and the average degree of these genes was 39.1, which measured the average number of direct connections between each member of NAGenes and other genes included in the PPI network, while, for the alcohol genes, 304 of the 316 genes could be mapped onto the human PPI network, with an average degree of 52.9 and for the cancer genes, 488 of the 519 genes could be mapped onto the human PPI network, with an average degree of 59.8. In order to have a more intuitive understanding of the degree characteristics, we plotted the degree distributions of the three gene sets (Figure 1). As shown, for all the three gene sets, the degrees scattered in a rather large range from 1 to more than 500. But the degree distributions were right-skewed, that is, the majority of the genes had only a few connections with other genes and a small number of genes had a large number of connections. Compared with the NAGenes, the average degree of the alcohol genes appeared to be closer to the cancer genes, but statistical test indicated that significant difference existed between the degrees of all the three gene sets (alcohol genes versus cancer genes, *P* = 1.93 × 10^−7^; alcohol genes versus NAGenes, *P* = 0.0031, Wilcoxon rank sum test). The degree distribution of NAGenes was also significantly different from that of both alcohol genes and cancer genes (NAGenes versus alcohol genes, *P* = 0.0031; NAGenes versus cancer genes, *P* = 1.93 × 10^−13^, Wilcoxon rank sum test). But compared with the cancer genes, the NAGenes and the alcohol genes tended to have lower or moderate connections, for example, 67% and 54% of the NAGenes, and the alcohol genes fell in the degree interval of 1–20, respectively, while only 37% of the cancer genes were included in this range (Figure 2). A close check of the degree of NAGenes showed that genes with more specific functions, such as those related to synaptic transmission (e.g., neuronal acetylcholine receptor subunit alpha-1 [CHRNA1], CHRNA2, CHRNB1, and CHRNB2), drug metabolism (e.g., N-acetyltransferase 2 [NAT2], tryptophan hydroxylase 2 [TPH2], and cytochrome P450 2A6 [CYP2A6]), and transport (e.g., solute carrier family 9 member 9 [SLC9A9], solute carrier organic anion transporter family member 3A1 [SLCO3A1], and solute carrier family 1 member 2 [SLC1A2]), tended to have smaller degrees, while the genes expressed in a large range of cell types/tissues or involved in broad physiological processes were more likely to larger degrees, for example, nuclear receptor subfamily 3 group C member 1 (NR3C1), beta-2 adrenergic receptor (ADRB2), estrogen receptor alpha (ESR1), and tumor protein p53 (TP53). Thus, although all the members of NAGenes may be nicotine addiction-related, those with smaller degrees are more likely to be involved in biological processes or neuronal activities invoked by nicotine.

**Figure 3:**
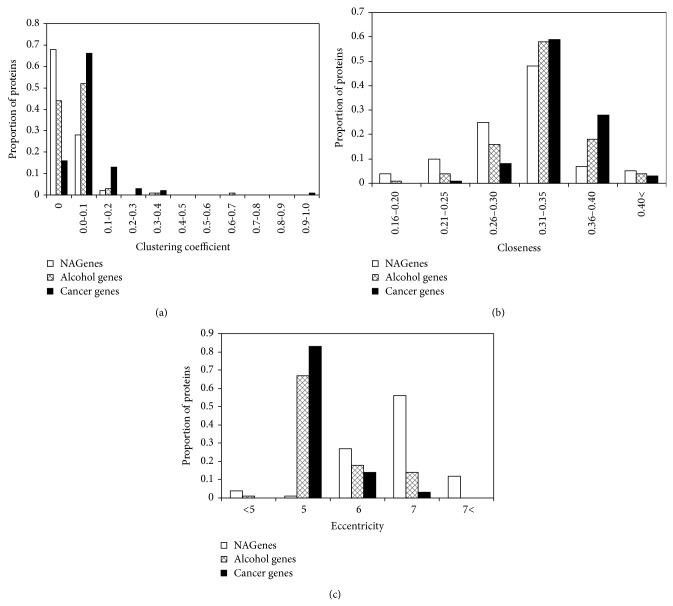
Topological measures distribution of NAGenes, alcohol genes, and cancer genes. *y*-axis represents the proportion of proteins having a specific measurement. (a) Clustering coefficient. (b) Closeness. (c) Eccentricity.

Clustering coefficient measures the interconnectivity of neighboring genes in a network. Generally, a gene with larger clustering coefficient has a higher density of network connection. The average clustering coefficients of NAGenes, alcohol genes, and cancer genes were 0.02, 0.03, and 0.06, respectively. To better describe the characteristics of the clustering coefficient, we summarized them using histogram with an interval of 0.1 (Figure 3(a)). Among the three gene sets, the proportion of genes with clustering coefficient of 0 was much higher for NAGenes (67.8%) than the alcohol genes (44.1%) and cancer genes (16.0%). Within the intervals 0-0.1, the proportion of NAGenes included was 96.2%, which is higher than the other two gene sets (alcohol: 95.7%; cancer: 81.6%). Interestingly, when the clustering coefficient was greater than 0.4, the proportion of NAGenes was 0. Thus, NAGenes were likely to be less connected with each other than the alcohol genes or the cancer genes. In addition, we also analyzed the distribution of closeness and eccentricity of the NAGenes in the human PPI network. Usually, a gene with higher closeness is more likely to be a central gene in the network, and a gene with larger eccentricity is closer to the fringe of the network [46, 47].  Figure 3(b) showed that NAGenes had a smaller closeness compared with the alcohol genes or the cancer genes, but the eccentricity distribution of NAGenes showed an opposite trend, following a more right-skewed distribution (Figure 3(c)). These results revealed that the NAGenes may be less central in the PPI network compared with the other two gene sets.

### 3.2. Cluster Analysis within the Global Network of NAGenes

Besides characterizing the interaction networks with respect to their topological features, the biological network can also be clustered or partitioned into modules, which provides an insight into the overall organization of the relationship within the PPI network [32]. Clustering algorithms have previously been shown to be useful in predicting the molecular modules that participate in similar biological process.

**Figure 4:**
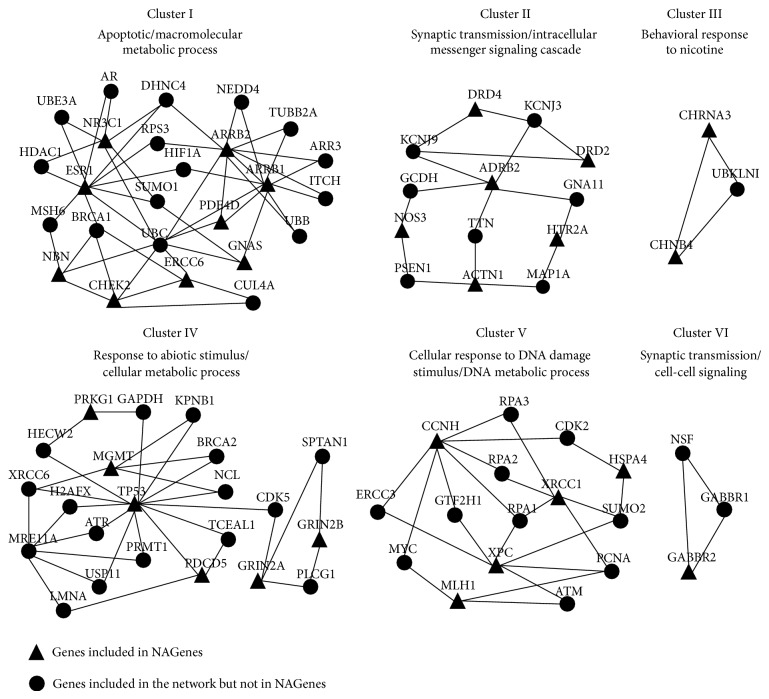
Gene clusters identified by MCODE. NAGenes are shown as triangular nodes and non-NAGenes are ellipse nodes. The functional descriptors of each cluster are based on Gene Ontology term.

By using the clustering algorithm to the network associated with nicotine addiction, we identified 6 clusters including 81 nodes (genes in our case) and 126 edges. Out of these nodes, 30 (37.04%) were included in the 208 genes mapping into the human PPI network. These clusters were ranked according to their density and the number of proteins (genes) included (Table 1 and Figure 4). As shown, the clusters were involved in multiple biological functional categories. For example, the majority of the genes in cluster I were associated with apoptotic and macromolecular metabolic process. Three genes associated with nicotine addiction, estrogen receptor 1 (ESR1), arrestin beta 1 (ARRB1), and ARRB2, were located close to the center of this cluster (Figure 4). ESR1, as the specific nuclear receptor of sex hormones, widely distributes in the dopaminergic midbrain neurons and is able to modulate the neurotransmitter systems of the brain reward circuitry [48]. Moreover, ESR1 also plays an important role in apoptotic process. ARRB1 and ARRB2 are ubiquitous scaffolding proteins. They can regulate multiple intracellular signaling proteins involved in cell proliferation and differentiation and have important roles in mitogenic and antiapoptotic function of nicotine [49, 50]. The overall functional theme of Clusters II, III, and VI was synaptic transmission. Dopamine receptor D2 (DRD2) and DRD4 are both dopamine receptors that are critical for the reinforcing effects or rewarding behaviors of nicotine [51, 52]. GABA B receptor 1 (GABBR1) and GABBR2, the two receptors of the major inhibitory neurotransmitter GABA, play important roles in the development of nicotine addiction [53].

Each cluster also contained genes not included in NAGenes (Figure 4). A close inspection showed that some of these additional genes were potentially related to nicotine addiction. For example, N-ethylmaleimide-sensitive factor (NSF) [54], ubiquitin b (UBB) [55], small ubiquitin-related modifier 2 (SUMO2) [55], cyclin-dependent kinase 5 (CDK5) [56], and phospholipase C gamma 1 (PLCG1) [57] have been reported to be associated with nicotine addiction or regulated by nicotine exposure. Thus, further exploration on the genes included in these clusters may help us to identify more nicotine addiction-related candidate genes.

### 3.3. Functional Annotation Analysis

**Figure 5:**
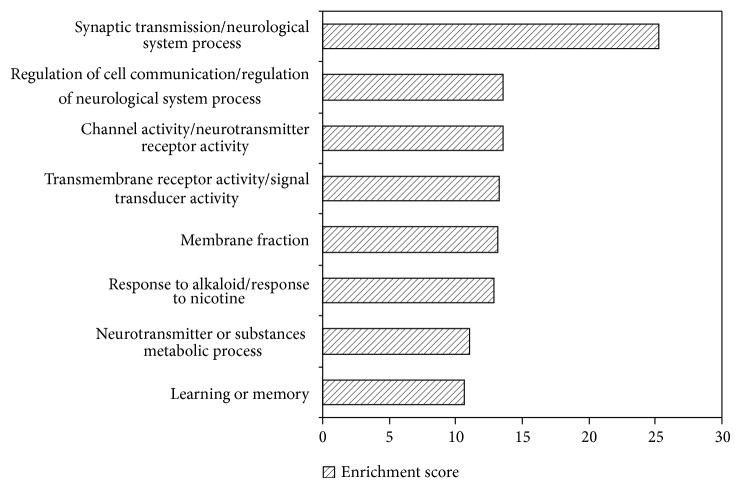
Enriched functional annotation in NAGenes (enrichment score > 10). Detailed information can be seen in supplementary Table  S1 in Supplementary Material available online at http://dx.doi.org/10.1155/2015/313709.

To obtain a more systematic view of the biological function of the genes involved in nicotine addiction, we performed functional enrichment analysis on NAGenes. In earlier study, a preliminary functional annotation analysis showed that genes related to biological processes like neurodevelopment and signal transduction were overrepresented in NAGenes [35]. Here, we provided a more comprehensive exploration on the function features of these genes. For the 220 genes, 73 annotation clusters were identified in the candidate genes (enrichment score > 1.3). Of these annotation clusters, eight clusters with enrichment scores higher than 10 were displayed with the representative GO terms (Figure 5 and Table  S1). From a wide view of the annotation clusters, functional annotations associated with neurodevelopment and neurotransmitters were significantly overrepresented in the NAGenes. In the top two annotation clusters (Clusters 1 and 2), eight terms, including transmission of nerve impulse (FDR = 1.85 × 10^−28^), synaptic transmission (FDR = 3.32 × 10^−28^), system process (FDR = 3.84 × 10^−19^), and neurological system process (FDR = 2.76 × 10^−18^), were directly related to neurodevelopment, consistent with the previous reports that there is a relationship between the pathology of nicotine addiction and the development of neuron system. Moreover, the majority of terms in Cluster 3 were associated with neurotransmitter receptor or channel activity, for example, extracellular ligand-gated ion channel activity (FDR = 2.28 × 10^−19^), neurotransmitter receptor activity (FDR = 5.80 × 10^−18^), and acetylcholine receptor activity (FDR = 6.06 × 10^−18^) (Table S1). These results indicated the importance of neurotransmitters and related molecules in the development of nicotine addiction. Importantly, we found that calcium ion transport (FDR = 0.02) was also overrepresented in the candidate genes, consistent with the reports that the ligand-gated cation channels play an important role in regulating various neuronal activities by mediating intracellular Ca^2+^ concentration, including neurotransmitter release [58, 59]. In Cluster 7, the overall functional theme was various neurotransmitter or substances metabolic process, such as dopamine metabolic process (FDR = 1.76 × 10^−12^), catecholamine metabolic process (FDR = 6.58 × 10^−11^), diol metabolic process (FDR = 6.58 × 10^−11^), and cellular amino acid derivative metabolic process (FDR = 5.21 × 10^−6^). These metabolic processes had important roles not only in the development of nicotine addiction, but also in the harm to human health. In addition, Cluster 8 was concentrated on learning or memory, which reflected a kind of pathological forms of nicotine addiction. In summary, the molecular mechanisms underlying nicotine addiction are extremely complex in that they involve many genes and biological functions. Through its direct or indirect interactions with these genes, nicotine can regulate various physiological processes, such as learning and memory, synaptic function, response to stress, and addiction [60–63]. Our results also demonstrated that functional annotation cluster analysis can provide useful insights for intuitive understanding of addiction mechanisms. Furthermore, as neurodevelopment system and neuronal signaling cascades in the brain play important roles in the pathology of nicotine addiction, the genes and pathways related to these biological processes should be the major targets in nicotine addiction study.

## 4. Conclusions

To achieve better understanding of the molecular mechanisms underlying nicotine addiction, it is necessary to adopt a system biology frame to analyze the candidate genes related to nicotine addiction. In this study, we explored the global network topological characteristics of nicotine addiction. The results revealed that the topological features of NAGenes were significantly different from alcohol genes and cancer genes. Specifically, NAGenes tended to have a more moderate degree and weaker clustering coefficient and they were likely to be in the network margin. Further, integrating the information from the functional modules identified in the global network and annotation cluster analysis, we found that nicotine addiction was involved in many biological functions, such as neurodevelopment, neurotransmitters activity, and various metabolic processes. Our preliminary results present a wealth of potential functional information underlying the mechanism of nicotine addiction and they are valuable for further investigation.

## Supplementary Material

Gene Ontology (GO) annotation was used to explore the function features of the nicotine addiction-related genes via The Database for Annotation, Visualization and Integrated Discovery (DAVID; http://david.abcc.ncifcrf.gov/). For the 220 genes, eight clusters with enrichment scores higher than 10 were identified by DAVID, and the representative GO terms in each cluster were displayed in the Supplemental Table S1.

## Figures and Tables

**Table 1:** Gene clusters identified in the nicotine addiction-related network.

Cluster	Cluster function	Score^a^	Nodes	Edges	Gene symbol
I	Apoptotic/macromolecular metabolic process	4.08	25	49	ARRB2, ARRB1, CUL4A, HDAC1, RPS3, ERCC6, GNAS, UBE3A, NBN, CHEK2, BRCA1, ESR1, ARR3, AR, HDAC2, NEDD4, UBB, MSH6, NR3C1, UBC, PDE4D, SUMO1, HIF1A, TUBB2A, ITCH

II	Synaptic transmission/intracellular and second messenger signaling cascade	2.67	13	16	KCNJ9, DRD2, ADRB2, DRD4, NOS3, MAP1A, GCDH, TTN, HTR2A, PSEN1, ACTN1, KCNJ3, GNA11

III	Behavioral response to nicotine	3.00	3	3	UBQLN1, CHRNB4, CHRNA3

IV	Response to abiotic stimulus/cellular metabolic process	3.05	22	32	BRCA2, GAPDH, H2AFX, SPTAN1, PRMT1, PRKG1, MGMT, NCL, HECW2, USP11, ATR, LMNA, GRIN2A, CDK5, TP53, GRIN2B, KPNB1, XRCC6, MRE11A, TCEAL1, PLCG1, PDCD5

V	Cellular response to DNA damage stimulus/DNA metabolic process	3.29	15	23	RPA2, RPA3, CCNH, HSPA4, ERCC3, XRCC1, RPA1, GTF2H1, MLH1, PCNA, MYC, XPC, ATM, CDK2, SUMO2

VI	Synaptic transmission/cell-cell signaling	3.00	3	3	NSF, GABBR1, GABBR2

^a^Score is defined as the product of the cluster density and the number of vertices (proteins) in the cluster (DC × |*V*|).
