# Supplementary material for: A Systematic Analysis of Candidate Genes Associated with Nicotine Addiction
Source: Biomed Res Int. 2015 May 4;2015:313709. doi: 10.1155/2015/313709 (PMC4434171; doi:10.1155/2015/313709)
Supplement: Supplementary file 1 — Gene Ontology (GO) annotation was used to explore the function features of the nicotine addiction-related genes via The Database for Annotation, Visualization and Integrated Discovery (DAVID; http://david.abcc.ncifcrf.gov/). For the 220 genes, eight clusters with enrichment scores higher than 10 were identified by DAVID, and the representative GO terms in each cluster were displayed in the Supplemental Table S1. [file 313709.f1.doc]

**Table S1. GO terms functional annotation according to DAVID analysis (enrichment score>=10).**

| **Category** | **Term** | **No. genes included** | **P-value** | **FDR** |
| --- | --- | --- | --- | --- |
| **Annotation Cluster 1** | **Enrichment Score: 25.29** |  |  |  |
|  | GO:0019226~transmission of nerve impulse | 48 | 1.05×10^-31^ | 1.85×10^-28^ |
|  | GO:0007268~synaptic transmission | 45 | 1.88×10^-31^ | 3.32×10^-28^ |
|  | GO:0007267~cell-cell signaling | 52 | 6.95×10^-25^ | 1.23×10^-21^ |
|  | GO:0007154~cell communication | 58 | 3.69×10^-24^ | 6.52×10^-21^ |
|  | GO:0003008~system process | 76 | 2.17×10^-22^ | 3.84×10^-19^ |
|  | GO:0050877~neurological system process | 67 | 1.57×10^-31^ | 2.76×10^-18^ |
| **Annotation Cluster 2** | **Enrichment Score: 13.57** |  |  |  |
|  | GO:0044057~regulation of system process | 32 | 2.80×10^-17^ | 4.95×10^-14^ |
|  | GO:0031644~regulation of neurological system process | 24 | 6.31×10^-17^ | 1.99×10^-13^ |
|  | GO:0051049~regulation of transport | 36 | 2.17×10^-16^ | 3.88×10^-13^ |
|  | GO:0032879~regulation of localization | 42 | 2.43×10^-16^ | 3.88×10^-13^ |
|  | GO:0051969~regulation of transmission of nerve impulse | 22 | 4.63×10^-15^ | 8.22675E-12 |
|  | GO:0050804~regulation of synaptic transmission | 21 | 1.21×10^-14^ | 2.14×10^-11^ |
|  | GO:0051239~regulation of multicellular organismal process | 48 | 1.02×10^-13^ | 1.80×10^-10^ |
|  | GO:0010646~regulation of cell communication | 42 | 7.39×10^-9^ | 1.30×10^-5^ |
| **Annotation Cluster 3** | **Enrichment Score: 13.52** |  |  |  |
|  | GO:0043176~amine binding | 35 | 5.74×10^-37^ | 8.43×10^-34^ |
|  | GO:0045211~postsynaptic membrane | 30 | 3.83×10^-27^ | 5.14×10^-24^ |
|  | GO:0042165~neurotransmitter binding | 26 | 1.98×10^-24^ | 2.92×10^-21^ |
|  | GO:0042166~acetylcholine binding | 16 | 4.39×10^-24^ | 6.46×10^-21^ |
|  | GO:0005892~nicotinic acetylcholine-gated receptor-channel complex | 14 | 1.46×10^-22^ | 1.96×10^-19^ |
|  | GO:0005230~extracellular ligand-gated ion channel activity | 22 | 1.55×10^-22^ | 2.28×10^-19^ |
|  | GO:0030594~neurotransmitter receptor activity | 23 | 3.95×10^-21^ | 5.80×10^-18^ |
|  | GO:0015464~acetylcholine receptor activity | 14 | 4.12×10^-21^ | 6.06×10^-18^ |
|  | GO:0015276~ligand-gated ion channel activity | 24 | 1.85×10^-19^ | 2.71×10^-16^ |
|  | GO:0030054~cell junction | 38 | 3.02×10^-17^ | 4.05×10^-14^ |
|  | GO:0022838~substrate specific channel activity | 31 | 3.98×10^-14^ | 5.85×10^-11^ |
|  | GO:0007271~synaptic transmission, cholinergic | 9 | 6.07×10^-12^ | 1.07×10^-8^ |
|  | GO:0035094~response to nicotine | 9 | 4.72×10^-11^ | 8.34×10^-8^ |
|  | GO:0015075~ion transmembrane transporter activity | 36 | 1.04×10^-10^ | 1.53×10^-7^ |
|  | GO:0022891~substrate-specific transmembrane transporter activity | 37 | 9.38×10^-10^ | 1.37×10^-6^ |
|  | GO:0022857~transmembrane transporter activity | 38 | 3.03×10^-9^ | 4.45×10^-6^ |
|  | GO:0006811~ion transport | 36 | 3.06×10^-9^ | 5.40×10^-6^ |
|  | GO:0051179~localization | 82 | 7.47×10^-9^ | 1.31×10^-5^ |
|  | GO:0051234~establishment of localization | 68 | 4.05×10^-6^ | 0.001 |
|  | GO:0006816~calcium ion transport | 12 | 9.24×10^-6^ | 0.02 |
| **Annotation Cluster 4** | **Enrichment Score: 13.23** |  |  |  |
|  | GO:0031226~intrinsic to plasma membrane | 67 | 3.14×10^-24^ | 4.20×10^-21^ |
|  | GO:0005887~integral to plasma membrane | 66 | 5.23×10^-24^ | 7.02×10^-21^ |
|  | GO:0044459~plasma membrane part | 89 | 1.20×10^-23^ | 1.61×10^-20^ |
|  | GO:0004872~receptor activity | 71 | 7.17×10^-16^ | 9.76×10^-13^ |
|  | GO:0005886~plasma membrane | 104 | 1.41×10^-15^ | 1.93×10^-12^ |
|  | GO:0004871~signal transducer activity | 78 | 7.88×10^-15^ | 1.15×10^-11^ |
|  | GO:0060089~molecular transducer activity | 78 | 7.88×10^-15^ | 1.15×10^-11^ |
|  | GO:0004888~transmembrane receptor activity | 54 | 5.74×10^-13^ | 8.43×10^-10^ |
|  | GO:0016020~membrane | 133 | 5.15×10^-7^ | 6.92×10^-4^ |
|  | GO:0044425~membrane part | 120 | 6.58×10^-6^ | 8.82×10^-3^ |
| **Annotation Cluster 5** | **Enrichment Score: 13.15** |  |  |  |
|  | GO:0000267~cell fraction | 51 | 3.50×10^-15^ | 4.76×10^-12^ |
|  | GO:0005626~insoluble fraction | 43 | 5.88×10^-14^ | 7.89×10^-11^ |
|  | GO:0005624~membrane fraction | 40 | 1.75×10^-12^ | 2.35×10^-9^ |
| **Annotation Cluster 6** | **Enrichment Score: 12.85** |  |  |  |
|  | GO:0014070~response to organic cyclic substance | 22 | 7.30×10^-17^ | 1.99×10^-13^ |
|  | GO:0043279~response to alkaloid | 14 | 8.29×10^-13^ | 1.46×10^-9^ |
|  | GO:0035094~response to nicotine | 9 | 4.72×10^-11^ | 8.34×10^-8^ |
| **Annotation Cluster 7** | **Enrichment Score: 11.00** |  |  |  |
|  | GO:0042417~dopamine metabolic process | 12 | 9.46×10^-16^ | 1.76×10^-12^ |
|  | GO:0009712~catechol metabolic process | 13 | 3.73×10^-14^ | 6.58×10^-11^ |
|  | GO:0034311~diol metabolic process | 13 | 3.73×10^-14^ | 6.58×10^-11^ |
|  | GO:0006584~catecholamine metabolic process | 13 | 3.73×10^-14^ | 6.58×10^-11^ |
|  | GO:0018958~phenol metabolic process | 13 | 5.61×10^-14^ | 9.91×10^-11^ |
|  | GO:0006725~cellular aromatic compound metabolic process | 20 | 1.49×10^-13^ | 2.64×10^-10^ |
|  | GO:0006575~cellular amino acid derivative metabolic process | 20 | 5.74×10^-12^ | 1.01×10^-8^ |
|  | GO:0006519~cellular amino acid and derivative metabolic process | 24 | 2.95×10^-9^ | 5.21×10^-6^ |
|  | GO:0006066~alcohol metabolic process | 23 | 4.78×10^-7^ | 0.84×10^-3^ |
|  | GO:0009308~amine metabolic process | 22 | 6.05×10^-7^ | 0.001 |
| **Annotation Cluster 8** | **Enrichment Score: 10.65** |  |  |  |
|  | GO:0007611~learning or memory | 20 | 3.21×10^-15^ | 5.68×10^-12^ |
|  | GO:0007612~learning | 14 | 3.57×10^-12^ | 6.31×10^-9^ |
|  | GO:0007613~memory | 11 | 1.95×10^-10^ | 3.45×10^-7^ |
